# Emergence of *Capnocytophaga canimorsus* and *Capnocytophaga cynodegmi* in oral cavities of newborn puppies, a pilot study

**DOI:** 10.1186/s13028-024-00751-z

**Published:** 2024-07-02

**Authors:** Kristiina Suominen, Silja Åvall-Jääskeläinen, Inka Sallinen, Anna-Maija Virtala, Joanna Koort

**Affiliations:** 1 Department of Veterinary Biosciences, Faculty of Veterinary Medicine, University of Helsinki, Helsinki, Finland; 2 Espoo Animal Hospital, IVC Evidensia, Espoo, Finland

**Keywords:** *Capnocytophaga*, Dogs, Oral microbiota, PCR, Zoonoses

## Abstract

*Capnocytophaga canimorsus* and *Capnocytophaga cynodegmi* are commensal bacteria in the oral cavities of dogs. Both are zoonotic pathogens that could infect humans via dog bites. *C. canimorsus* may cause life-threatening infections in humans, whereas *C. cynodegmi* infections tend to be milder and more localized. Capsular serovars A–C of *C. canimorsus* seem to be virulence-associated. Some of the *C. canimorsus* serovars described to date can also be detected in other *Capnocytophaga* species, including *C. cynodegmi.* The objective of this pilot study was to investigate the emergence of *C. canimorsus* and *C. cynodegmi* after birth in oral cavities of puppies and to evaluate the impact of the dam’s *Capnocytophaga* spp. carrier status on the emergence. Ten litters, altogether 59 puppies, were included in the study. The puppies and their dams were sampled at five time points over seven weeks after whelping. Oral swab samples taken were investigated for the presence of *C. canimorsus* and *C. cynodegmi* by species-specific polymerase chain reaction (PCR), the specificity of which was verified by sequencing a selection of the PCR products. Samples that were positive in *Capnocytophaga* PCR reactions were also capsular-typed by PCR to gain more knowledge about the *Capnocytophaga* spp. present in the samples. Altogether 10.2% and 11.9% of puppies, or 20.0% and 30.0% of litters tested PCR-positive for *C. canimorsus* and *C. cynodegmi*, respectively. *Capnocytophaga* PCR-positive puppy samples were always positive for only *C. cynodegmi* or *C. canimorsus*, not both. Most *Capnocytophaga* PCR-positive puppies became positive at the age of 5 to 7 weeks. Only a minority (5/16) of the *C. cynodegmi* PCR-positive dog samples were positive in capsular typing PCR, whereas all *C. canimorsus* PCR-positive dog samples were negative in capsular typing PCR. For all *Capnocytophaga* PCR-positive puppies, their dam was positive for the same *Capnocytophaga* species. These results suggest that puppies become colonized by *C. cynodegmi* or *C. canimorsus* from their dams at the time of deciduous teeth eruption.

**Supplementary Information:**

The online version contains supplementary material available at 10.1186/s13028-024-00751-z.

## Findings

The bacteria in the genus *Capnocytophaga* (family *Flavobacteriaceae*) are Gram-negative, capnophilic and belong to the oral microbiota of humans and animals [1]. In the oral microbiota of dogs, *Capnocytophaga canimorsus* and *Capnocytophaga cynodegmi* are the most commonly encountered *Capnocytophaga* species [2, 3]. Both species are zoonotic pathogens that could cause infections of varying severity in humans. *C. canimorsus* causes severe infections characterized by sepsis, whereas *C. cynodegmi* typically causes skin and soft tissue infections [4]. In *C. canimorsus*, three (namely A–C) of the currently described capsular serovars seem to be virulence-associated, as they cause most (about 90%) of the human *C. canimorsus* infections [5, 6]. The serovars described to date are not, however, specific to *C. canimorsus*, as some of them can be detected also in other *Capnocytophaga* species, namely in *C. cynodegmi* and *C. canis* [7], the latter of which is also a zoonotic pathogen [8]. The virulence potential for isolates belonging to these two species cannot, however, yet be linked to any specific serotype(s) [7].

Zoonotic *Capnocytophaga* infection may result from dog contact such as a bite or licking [4]. In dogs, the *Capnocytophaga* spp. have previously been detected in tooth plaque [3], oral swab samples [2], samples taken from incisor teeth and gingival margin [9] and saliva samples from gingival and cheek mucosa [10]. However, it is currently unknown when *Capnocytophaga* spp. first appear in the oral microbiota of dogs. This pilot study aimed to investigate by polymerase chain reaction (PCR) the emergence of *C. canimorsus* and *C. cynodegmi* in the oral cavities of puppies and to evaluate the impact of the dam’s *Capnocytophaga* spp. carrier status on the emergence of *Capnocytophaga* spp. in the oral microbiota of their puppies. Capsular PCR typing was also applied. We hypothesized that the *Capnocytophaga* spp. start emerging in the oral cavities of puppies after teeth eruption has begun, and that the dam would be the source of *Capnocytophaga* spp. to its puppies.

Privately owned dogs were recruited to this study via several breed association channels, including breed association websites. For practical reasons, we aimed for ten litters ending recruitment after the target number was reached. The breeds of dogs in this study were Shetland Sheepdog (*n* = 4), Flat-coated Retriever (*n* = 3), Labrador Retriever (*n* = 1), Golden Retriever (*n* = 1) and Bearded Collie (*n* = 1). Samples for this study were taken from 10 dams and their 59 puppies between March 2018 and June 2019. The breeders took all samples according to the written, video and verbal instructions provided (Additional file 1). Despite the detailed sampling instructions provided, aiming for the correct sampling area could have been challenging for the newborn puppies. None of the breeders, however, reported of such difficulties. Puppy samples were collected postnatally on weeks 0, 1, 3, 5 and 7, hereafter called the sampling period. The first sample was taken during the first 24 h after birth. For the dams, the samples were collected at same five time points as for the puppies.

Samples from the dams and puppies were taken by using sterile viscose-tipped swabs provided with Amies clear transport medium (Technical Service Consultants Ltd., Lancashire, UK). The swabs were rubbed against the gingiva or mucosa of the oral cavity, at the location of the canine teeth, for a few seconds. After sampling, the swabs were stored at room temperature and were examined within 0–3 days of sampling. There were some deviations from the sampling plan. The first samples from litter 7 and litter 10 could not be taken due to scheduling reasons. In litter 4, one puppy died on the third day after birth and was therefore only sampled once. From litter 10, in the last sampling only three puppies from the original eight were still at the breeder for sampling. In the laboratory, the swabs were suspended in 10 mL of brain heart infusion, porcine broth (Becton Dickinson and Company, Sparks, MD, USA) supplemented with 0.05% (wt/vol) L-cysteine HCl monohydrate (Sigma-Aldrich Co., St. Louis, MO, USA) and 0.25 mM iron (III) chloride hexahydrate (Sigma-Aldrich Co.) and cultured for 24 h at 37 °C in an aerobic atmosphere of 5% CO_2_.

Following incubation, the bacterial crude DNA was obtained and used as a template for PCR detection of *C. canimorsus* and C. *cynodegmi*, as described previously [2, 11]. If *C. canimorsus* or *C. cynodegmi* PCR-positive puppies were found in a litter, one puppy and one dam sample were sequenced for each such litter. Capsular typing by PCR was performed, as described previously [5], on dam and puppy samples that were positive in *Capnocytophaga* PCR reactions. A detailed description of the PCR methods, PCR product sequencing and statistical analyses performed in this study is provided in Additional File 2.

Altogether 10.2% of puppies tested PCR-positive for *C. canimorsus*, whereas 80.0% of the dams were positive in this PCR (Table 1). In addition, *C. cynodegmi* DNA was detected in 11.9% of the puppies, whereas 90.0% of the dams were positive for *C. cynodegmi* (Table 2). All *Capnocytophaga* PCR-positive puppies were positive in either *C. cynodegmi* PCR or *C. canimorsus* PCR, while the majority of dams (7/10) were positive in both PCRs. Most of the PCR-positive puppies became positive at the age of 5 to 7 weeks (Tables 1 and 2). At the end of our experiment, altogether 22.1% of the puppies were PCR-positive for *Capnocytophaga* (Tables 1 and 2). Sequencing of the selected *C. canimorsus* and *C. cynodegmi* PCR products confirmed their identity as *C. canimorsus* and *C. cynodegmi*, respectively, as the highest identities were always obtained for the respective type strain 16S rRNA gene sequences (results not shown). Sequencing also confirmed that the PCR products of a dam and its puppy were always either identical or nearly identical (99.26–100% identity; results not shown).

**Table 1:** Puppies’ and dams’ PCR-positivity for *C. canimorsus* at litter level

Litter	PCR result of dam	No. of puppies	Cumulative PCR-positivity of the puppies *N* (%, 95% CI^†^)					
			At 0 weeks	At 1 week	At 3 weeks	At 5 weeks	At 7 weeks	
1	Negative	3	0 (0.0, 0.0–56.2)	0 (0.0, 0.0–56.2)	0 (0.0, 0.0–56.2)	0 (0.0, 0.0–56.2)	0 (0.0, 0.0–56.2)	
2	**Positive**	10	0 (0.0, 0.0–27.8)	0 (0.0, 0.0–27.8)	0 (0.0, 0.0–27.8)	0 (0.0, 0.0–27.8)	0 (0.0, 0.0–27.8)	
3	Negative	2	0 (0.0, 0.0–65.8)	0 (0.0, 0.0–65.8)	0 (0.0, 0.0–65.8)	0 (0.0, 0.0–65.8)	0 (0.0, 0.0–65.8)	
4	**Positive**	13	0 (0.0, 0.0–22.8)	0 (0.0, 0.0–22.8)	0 (0.0, 0.0–22.8)	0 (0.0, 0.0–22.8)	0 (0.0, 0.0–22.8)	
5	**Positive**	3	0 (0.0, 0.0–56.2)	0 (0.0, 0.0–56.2)	0 (0.0, 0.0–56.2)	0 (0.0, 0.0–56.2)	**3** **(100.0, 43.9–100.0)**	
6	**Positive**	4	0 (0.0, 0.0–49.0)	0 (0.0, 0.0–49.0)	0 (0.0, 0.0–49.0)	0 (0.0, 0.0–49.0)	0 (0.0, 0.0–49.0)	
7	**Positive**	6	- ^‡^	0 (0.0, 0.0–39.0)	0 (0.0, 0.0–39.0)	0 (0.0, 0.0–39.0)	0 (0.0, 0.0–39.0)	
8	**Positive**	3	0 (0.0, 0.0–56.2)	0 (0.0, 0.0–56.2)	0 (0.0, 0.0–56.2)	**1** **(33.3, 6.2–79.2)**	**3** **(100.0, 43.9–100.0)**	
9	**Positive**	7	0 (0.0, 0.0–35.4)	0 (0.0, 0.0–35.4)	0 (0.0, 0.0–35.4)	0 (0.0, 0.0–35.4)	0 (0.0, 0.0–35.4)	
10	**Positive**	8	- ^‡^	0 (0.0, 0.0–32.4)	0 (0.0, 0.0–32.4)	0 (0.0, 0.0–32.4)	0 (0.0, 0.0–32.4)	
In total	**8** **(80.0, 49.0–94.3)**	59	0 (0.0, 0.0–6.1)	0 (0.0, 0.0–6.1)	0 (0.0, 0.0–6.1)	**1** **(1.7, 0.3–9.0)**	**6** **(10.2, 4.7–20.5)**	

For puppies, the results are shown cumulatively; after a positive PCR result, the puppy is regarded as positive for the later sampling times as well. The bottom row displays the total cumulative proportion of positive puppies for each sampling time. For dams, the result is shown as positive / negative. The bottom row also displays the total number of positive dogs. All positive results are in bold

^†^ CI = Confidence interval

^‡^- Samples not taken

**Table 2:** Puppies’ and dams’ PCR-positivity for *C. cynodegmi* at litter level

Litter	PCR result of dam	No. of puppies	Cumulative PCR-positivity of puppies *N* (%, 95% CI†)				
			At 0 weeks	At 1 week	At 3 weeks	At 5 weeks	At 7 weeks
1	**Positive**	3	0 (0.0, 0.0–56.2)	0 (0.0, 0.0–56.2)	0 (0.0, 0.0–56.2)	0 (0.0, 0.0–56.2)	**3** **(100.0, 43.9–100.0)**
2	**Positive**	10	0 (0.0, 0.0–27.8)	0 (0.0, 0.0–27.8)	0 (0.0, 0.0–27.8)	0 (0.0, 0.0–27.8)	0 (0.0, 0.0–27.8)
3	**Positive**	2	0 (0.0, 0.0–65.8)	0 (0.0, 0.0–65.8)	0 (0.0, 0.0–65.8)	0 (0.0, 0.0–65.8)	0 (0.0, 0.0–65.8)
4	**Positive**	13	**1** **(7.7, 1.4–33.3)**	**1** **(7.7, 1.4–33.3)**	**1** **(7.7, 1.4–33.37)**	**1** **(7.7, 1.4–33.3)**	**3** **(23.1, 8.2–50.3)**
5	Negative	3	0 (0.0, 0.0–56.2)	0 (0.0, 0.0–56.2)	0 (0.0, 0.0–56.2)	0 (0.0, 0.0–56.2)	0 (0.0, 0.0–56.2)
6	**Positive**	4	0 (0.0, 0.0–49.0)	0 (0.0, 0.0–49.0)	0 (0.0, 0.0–49.0)	0 (0.0, 0.0–49.0)	**1** **(25.0, 4.6–69.9)**
7	**Positive**	6	- ^‡^	0 (0.0, 0.0–39.0)	0 (0.0, 0.0–39.0)	0 (0.0, 0.0–39.0)	0 (0.0, 0.0–39.0)
8	**Positive**	3	0 (0.0, 0.0–56.2)	0 (0.0, 0.0–56.2)	0 (0.0, 0.0–56.2)	0 (0.0, 0.0–56.2)	0 (0.0, 0.0–56.2)
9	**Positive**	7	0 (0.0, 0.0–35.4)	0 (0.0, 0.0–35.4)	0 (0.0, 0.0–35.4)	0 (0.0, 0.0–35.4)	0 (0.0, 0.0–35.4)
10	**Positive**	8	- ^‡^	0 (0.0, 0.0–32.4)	0 (0.0, 0.0–32.4)	0 (0.0, 0.0–32.4)	0 (0.0, 0.0–32.4)
In total	**9** **(90.0, 59.6–98.2)**	59	**1** **(2.2, 0.4–11.6)**	**1** **(1.7, 0.3–9.0**	**1** **(1.7, 0.3–9.0)**	**1** **(1.7, 0.3–9.0)**	**7** **(11.9, 5.9–22.5)**

For puppies, the results are shown cumulatively; after a positive PCR result, the puppy is regarded as positive for the later sampling times as well. The bottom row displays the total cumulative proportion of positive puppies for each sampling time. For dams, the result is shown as positive / negative. The bottom row also displays the total number of positive dogs. All positive results are in bold

^†^CI = Confidence interval

^‡^- Samples not taken

The frequency of capsular serovars ABC and serovar D by PCR in the dam and puppy samples is shown in Additional File 3. All positive capsular typing PCR results were obtained from dogs that were PCR-positive for *C. cynodegmi.* The only positive capsular PCR samples from puppies (all from the same litter and positive for serovar D) were obtained in samples taken at 7 weeks (results not shown) (Additional File 3).

For all the *Capnocytophaga* spp. PCR-positive puppies, their dam was positive for the same primer pair, and the puppy samples sequenced were identical or highly identical to the PCR product of their dam. Also, for all three capsular typing PCR-positive puppies, their dam was positive for the same capsular type. These results indicate that the puppies contract *Capnocytophaga* spp. from their dam, e.g. by licking the dam’s lips or by regurgitated food. However, it is also possible that the puppies got *Capnocytophaga* spp. from other dogs in the household since in all households there were other dogs besides the dam. We do not know how much these other dogs interacted with the puppies. It is unlikely that the puppies contracted *Capnocytophaga* spp. from the environment since no environmental sources for these bacteria are known.

Most of the *Capnocytophaga* spp. PCR-positive puppies were detected at the age of 5 or 7 weeks. Deciduous teeth start to erupt around 3 to 5 weeks of age [12], and thus, the results seem to support our hypothesis that *Capnocytophaga* spp. start establishing in the oral cavities of puppies after teeth eruption has begun. Teeth eruption creates more niches for bacterial colonization, for e.g. gingival pockets [13] and, in humans, the establishment of *Capnocytophaga* spp. has been suggested to be connected to teeth eruption [14–16]. However, further studies are needed to elucidate this matter in dogs.

In a previous study, prevalences of 74% for *C. canimorsus* and 86% for *C. cynodegmi* were reported in dogs, detected using the same PCR detection method as used in our study [2]. However, the ages of the dogs were not reported [2], which complicates the comparison with our study. While the proportions of *Capnocytophaga* spp. PCR-positive dams in our study (80% for *C. canimorsus* and 90% for *C. cynodegmi*) were in accordance with a previous study [2], the proportion of PCR-positive puppies in our study (average 22%) was noticeably lower. A culture-based survey study [3] noted that very few puppies less than 6 months of age had *Capnocytophaga* spp. (3/17, 18%), and *C. canimorsus* was even rarer (1/17, 6%), which are in line with our results. Although the results from bacterial culture-based methods cannot be directly compared with PCR-based methods, as PCR also detects nonviable bacteria, our findings clearly demonstrate that in puppies aged 0–7 weeks *Capnocytophaga* spp. are not as common as in their dams.

Our results indicate that puppies do not, to a significant extent, acquire *C. canimorsus* or *C. cynodegmi* at birth and *Capnocytophaga* spp. start establishing in the oral cavities of puppies mostly after teeth eruption has begun. The dam is the most likely source of *Capnocytophaga* spp. for her puppies. Further studies are, however, warranted due to the small sample size.

### Electronic supplementary material

Below is the link to the electronic supplementary material.

Additional file 1. Written sampling instructions for the breeders

Additional File 2. Supplementary methods description, including PCR assays, PCR product sequencing and statistical analyses

Additional File 3. Puppies’ and dams’ PCR-positivity in capsular ABC and D PCR

## Data Availability

The datasets used and analysed during this study are available from the corresponding author on reasonable request.

## Declarations

### Prior publication

Data have not been published previously.
