# Supplementary material for: Emergence of Capnocytophaga canimorsus and Capnocytophaga cynodegmi in oral cavities of newborn puppies, a pilot study
Source: Acta Vet Scand. 2024 Jul 2;66:26. doi: 10.1186/s13028-024-00751-z (PMC11218291; doi:10.1186/s13028-024-00751-z)
Supplement: Supplementary file 1 — Additional file 1. Written sampling instructions for the breeders [file 13028_2024_751_MOESM1_ESM.docx]

Additional file 1. Instructions for the breeders.

# Research on the development of the dog's oral bacterial flora

Written instructions for sampling

1. Open the packaging of the sampling kit and take the swab out of the kit, holding onto the blue part of the swab.

2. Take a sample from the dog's mouth. At each sampling time, take a sample of each puppy and dam. Take a sample from the outer surface of the gum at approximately the location of upper canine tooth.

o Rub and roll the cotton end of the swab against the gum for five seconds.

o Touch only the blue part of the swab and do not let the swab touch anything other than the gum in the dog's mouth.

3. After sampling, open the cap from the tube and insert the swab into the tube.

You can throw away the extra cap.

4. Type the dog's name/tag together with date and time of sampling on the side of the tube.

o Store the sample at room temperature.

o The sample must be received by the laboratory within 72 hours (i.e. 3 days) of sampling.

Sampling video on YouTube: <https://youtu.be/4h6V3XCdnPs>

If you have any questions, please contact the research personnel.
