# Supplementary material for: Emergence of Capnocytophaga canimorsus and Capnocytophaga cynodegmi in oral cavities of newborn puppies, a pilot study
Source: Acta Vet Scand. 2024 Jul 2;66:26. doi: 10.1186/s13028-024-00751-z (PMC11218291; doi:10.1186/s13028-024-00751-z)
Supplement: Supplementary file 2 — Additional File 2. Supplementary methods description, including PCR assays, PCR product sequencing and statistical analyses [file 13028_2024_751_MOESM2_ESM.docx]

Additional File 2. Supplementary methods description, including PCR assays, PCR product sequencing and statistical analyses

**Template preparation, PCR detection of *C. canimorsus* and C. *cynodegmi* and sequencing of PCR products**

The template for all PCRs of this study was prepared essentially as described previously [1,2]. To harvest bacterial cells from the 24 h brain heart broth cultures, 9 mL of the culture was centrifuged at 4 500 g RT for 10 min. The harvested cells were suspended in 1.35 mL of sterilized H_2_O, followed by heating at 100°C for 10 min. After heating, centrifugation at 14 000 g RT for 5 min was performed, and the supernatants containing bacterial DNA were collected for use in PCR experiments. Samples were stored at -20°C until the PCR assays were performed.

Primer pairs Cal2/CaR and Cal2/CyR were used to amplify a 427 base pair region of the 16S rRNA gene of *C. canimorsus* and *C. cynodegmi*, respectively. The specificity of these primer pairs for *C. canimorsus* and *C. cynodegmi* has been previously demonstrated [1]. Reaction mixes for both primer combinations mentioned above were conducted in a final volume of 25 µL, comprising 3 µL of the prepared template, 0.5 μM of each primer, 0.5 U of DyNAzyme II DNA polymerase (Thermo Fisher Scientific, Waltham, MA, USA; production discontinued) or 0.625 U of Dream Taq DNA polymerase (Thermo Fisher Scientific) and 200 µM of each dNTP (Thermo Fisher Scientific) in 1x reaction buffer supplied with the DNA polymerase in question. Most puppy samples were initially studied in Cal2/CyR PCR using pooled samples (three samples mixed; 5 µL of each sample), and 3 µL of this mix was used as a template). If a pooled puppy sample gave a positive PCR result, each of the puppy samples included in the mix was studied individually with the same primer pair. The functionality of the use of pooled puppy samples in PCRs with primer pair Cal2/CyR was initially verified using various dog oral swab samples (earlier tested individually to be either positive or negative in Cal2/CyR PCR) prepared like the puppy samples described earlier (results not shown). All dam samples were studied individually in Cal2/CyR PCR. PCR performed with primer pairs Cal2/CaR was always conducted using only individual puppy or dam samples as templates.

The PCR conditions in both primer combinations used were as follows: one cycle of denaturation at 95°C for 5 min, 35 cycles of denaturation at 95°C for 30 s, annealing at 58°C for 1 min, extension at 72°C for 1 min and final extension at 72°C for 7 min. A positive control (Cal2/CaR: *C. canimorsus* DSM 19204 DNA; Cal2/CyR: *C. cynodegmi* DSM 19736 DNA) and a no-template control were included in each run. PCR products were separated by gel electrophoresis on 1.2% (wt/vol) agarose gel at 80 V for 45 min and visualized by ethidium bromide under UV light.

Sequencing of selected *C. cynodegmi* (n=6) or *C. canimorsus* PCR (n=4) products was achieved at the DNA Sequencing and Genomics Laboratory at the Institute of Biotechnology, Helsinki, Finland. Sequencing was performed in both directions using the same primers as used for the initial PCR. Assembled sequences were compared with published sequences in GeneBank by blastn tool. The search was limited to the type material, otherwise default settings were used. Blastn was also used for pairwise comparisons between the PCR products of a dam and its puppy.

**Capsular ABC and D PCR**

Primers 8296/8297 were used in the seroABC PCR, allowing the detection of capsular serovars A, B and C in a single reaction. Because the product amplified from serovars A, B and C is of the same size, by this PCR alone a distinction between these three serotypes cannot be made. Primer pair 8276/8277 was used for the detection of capsular serovar D (seroD). Reaction mix composition was otherwise as detailed above in the description of *C. canimorsus* and *C. cynodegmi* PCR. Amplification conditions consisted of one cycle of denaturation at 95°C for 3 min, followed by 35 cycles of denaturation at 95°C for 30 s, annealing at 52°C for 45 s, extension at 72°C for 1 min 30 s (8296/8297) or 1 min (8276/8277) and final extension at 72°C for 7 min. A positive control (seroABC: *C. canimorsus* LMG 28512; seroD: *C. canimorsus* DSM 19204) and a no-template control were included in each run. PCR products were separated by gel electrophoresis and visualized as described above in the description of *C. canimorsus* and *C. cynodegmi* PCR.

**Statistical analyses**

Laboratory data were described using frequencies and percentages. Epitools online calculator [3] was used to calculate the 95% confidence intervals for percentages with the Wilson method [4].

**References**

1. Suzuki M, Kimura M, Imaoka K, Yamada A. Prevalence of *Capnocytophaga canimorsus* and *Capnocytophaga cynodegmi* in dogs and cats determined by using newly established species-specific PCR. Vet Microbiol. 2010;144:172-6.
2. Umeda K, Hatakeyama R, Abe T, Takakura K, Wada T, Ogasawara J, Sanada S, Hase A. Distribution of *Capnocytophaga canimorsus* in dogs and cats with genetic characterization of isolates. Vet Microbiol. 2014;17:153-9.
3. Sergeant, ESG. Epitools Epidemiological Calculators. Ausvet. 2018. <http://epitools.ausvet.com.au>. Accessed 31 Oct 2023.
4. Brown, LD, Cai TT and DasGupta A. Interval estimation for a binomial proportion. Stat Sci. 2001;16:101-33.
