# Supplementary material for: Emergence of Capnocytophaga canimorsus and Capnocytophaga cynodegmi in oral cavities of newborn puppies, a pilot study
Source: Acta Vet Scand. 2024 Jul 2;66:26. doi: 10.1186/s13028-024-00751-z (PMC11218291; doi:10.1186/s13028-024-00751-z)
Supplement: Supplementary file 3 — Additional File 3. Puppies’ and dams’ PCR-positivity in capsular ABC and D PCR [file 13028_2024_751_MOESM3_ESM.docx]

Additional File 3. Puppies’ and dams’ PCR-seropositivity in capsular ABC and D PCR.

| Litter | |  | PCR-seropositivity of dams and number  of PCR-seropositive puppies per litter | |  |
| --- | --- | --- | --- | --- | --- |
|  |  |  | **Serovars ABC** | **Serovar D** | |
| 1 | Dam | | **positive** | **positive** | |
|  | Puppies | | **0**/3 | **3**/3 | |
| 3 | Dam | | negative | **positive** | |
|  | Puppies | | **-^*^** | **-** | |
| 2,  4–10 | Dams | | negative | negative | |
|  | Puppies | | **0**/10 | **0**/10 | |

**^*^**- samples not studied
